# Supplementary figures and images for: Basal IFNλ2/3 signaling is required for ISG expression and viral control in human intestinal epithelial cells
Source: PLoS Pathog. 2026 Jan 12;22(1):e1013857. doi: 10.1371/journal.ppat.1013857 (PMC12822948; doi:10.1371/journal.ppat.1013857)

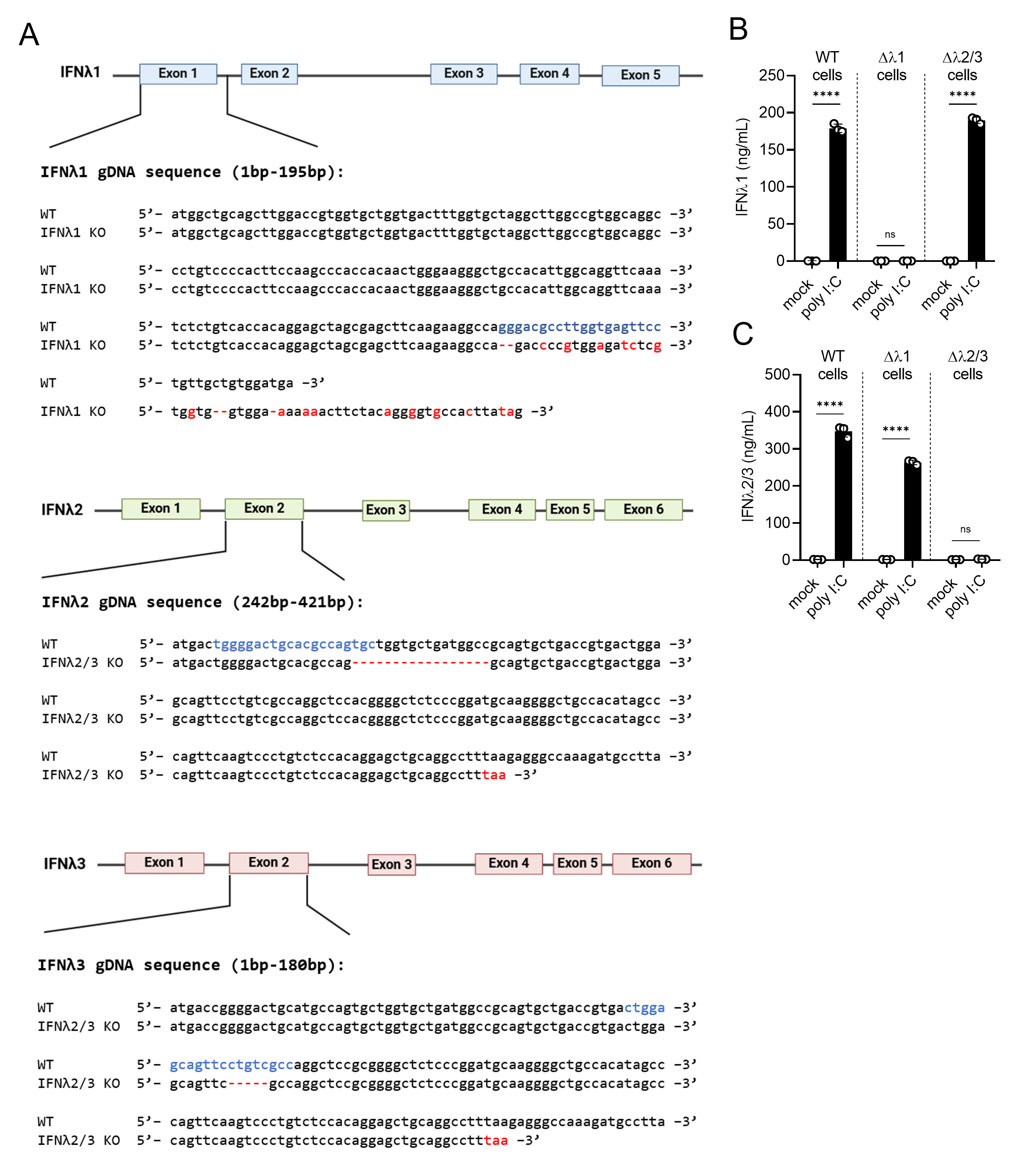

Supplement: S1 Fig — (A) Genomic DNA (gDNA) from T84 WT, IFNλ1 KO, and IFNλ2/3 KO cells were isolated, and PCR was performed for the amplification of IFNλ1, IFNλ2, and IFNλ3 loci. PCR products were sequenced by Sanger sequencing. The Basic Local Alignment Search Tool (BLAST) was used to compare the sequences. (B,C). T84 WT, IFNλ1 KO, and IFNλ2/3 KO cells were seeded in 48-well plates and transfected with poly I:C for 6 hours. Cell supernatants were collected and analyzed by ELISA to measure (B) IFNλ1 and (C) IFNλ2/3 protein levels. Data represent n ≥ 3 biological replicates. Statistical significance was determined using two-way ANOVA (P < 0.0001 ****, ns = not significant). Error bars represent standard deviation with the mean as the center. (TIF) [file ppat.1013857.s001.tif]

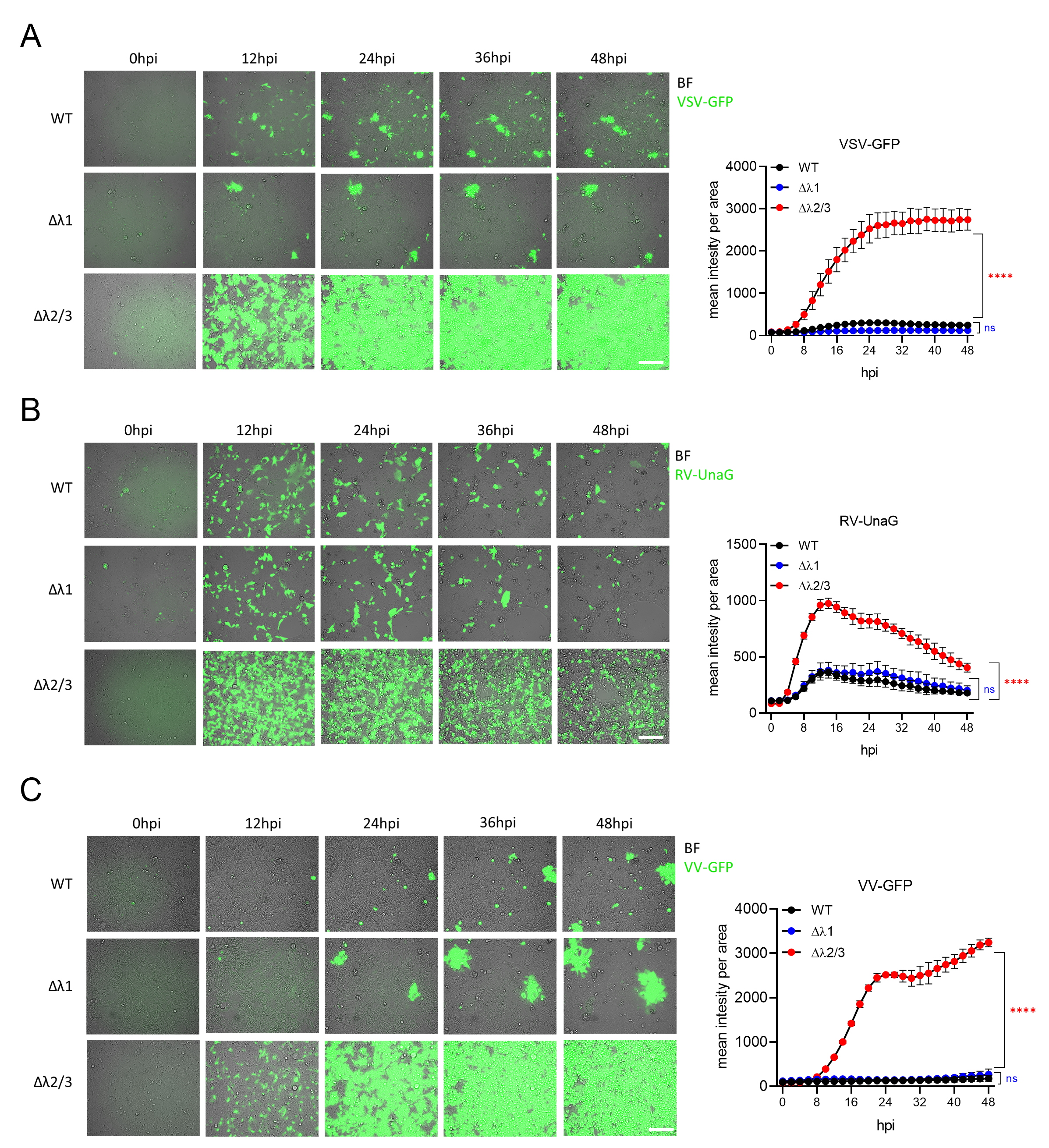

Supplement: S2 Fig — T84 WT, IFNλ1 KO, and IFNλ2/3 KO cells were seeded in 96-well plates and infected two days later with (A) VSV-GFP, (B) RV-UnaG, and (C) VV-GFP. Viral spread was monitored using live-cell microscopy every 2 hours for 48 hours. (left) Representative brightfield (gray) and fluorescence images show infection (green) at 0, 12, 24, 36, and 48 hpi. Representative images shown. Scale bar = 250 μm. (right) Mean fluorescence intensity over time per field of view was quantified using ImageJ Fiji. Data represent n ≥ 3 biological replicates. Statistical significance was determined using two-way ANOVA (P < 0.0001 ****, ns = not significant). Error bars represent standard deviation with the mean as the center. (TIF) [file ppat.1013857.s002.tif]

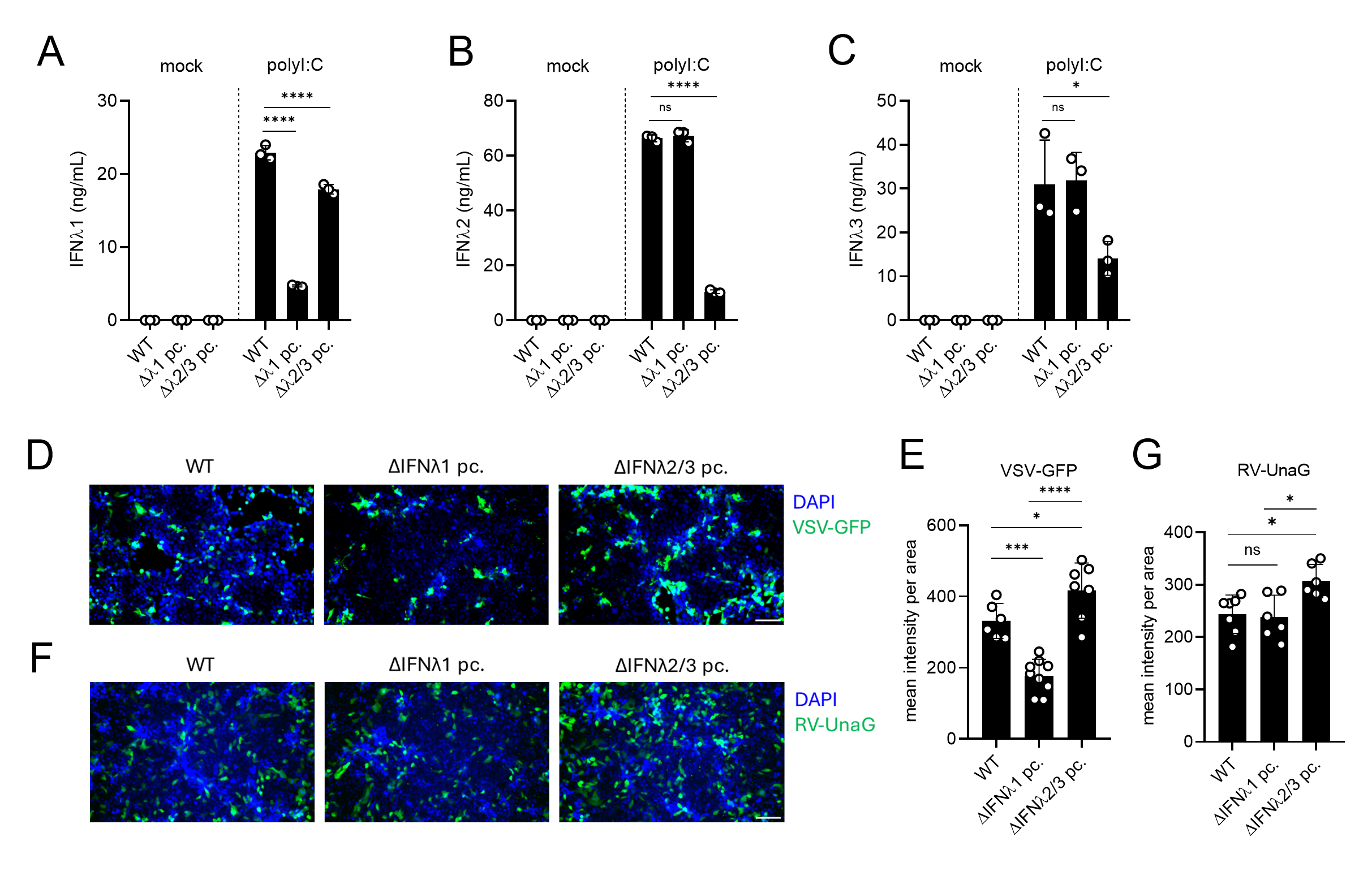

Supplement: S3 Fig — (A-C) T84 WT, IFNλ1 KO polyclonal (pc.), and IFNλ2/3 KO polyclonal cells (pc.) were seeded in 48-well plates, and next day, transfected with poly I:C for 6 hours. Cell supernatants were collected and analyzed by ELISA to measure (A) IFNλ1; (B) IFNλ2; (C) IFNλ3 protein levels. (D-G) T84 WT, IFNλ1 KO polyclonal (pc.), and IFNλ2/3 KO polyclonal (pc.) cells were seeded in 48-well plates. The following day, cells were infected with (D, E) VSV-GFP (MOI = 1) for 7 hours and (F, G) RV-UnaG (MOI = 1) for 16 hours. Nuclei were stained with Hoechst (blue), and infected cells are shown in green. (D, F) Representative images and (E, G) corresponding quantification (right) are shown for each virus. Scale bar = 100 μm. Data represent ≥3 independent biological replicates. Statistical significance was determined by one-way ANOVA (*P < 0.05, ***P < 0.001, ****P < 0.0001, ns = not significant). Error bars represent standard deviation with the mean as the center. (TIF) [file ppat.1013857.s003.tif]

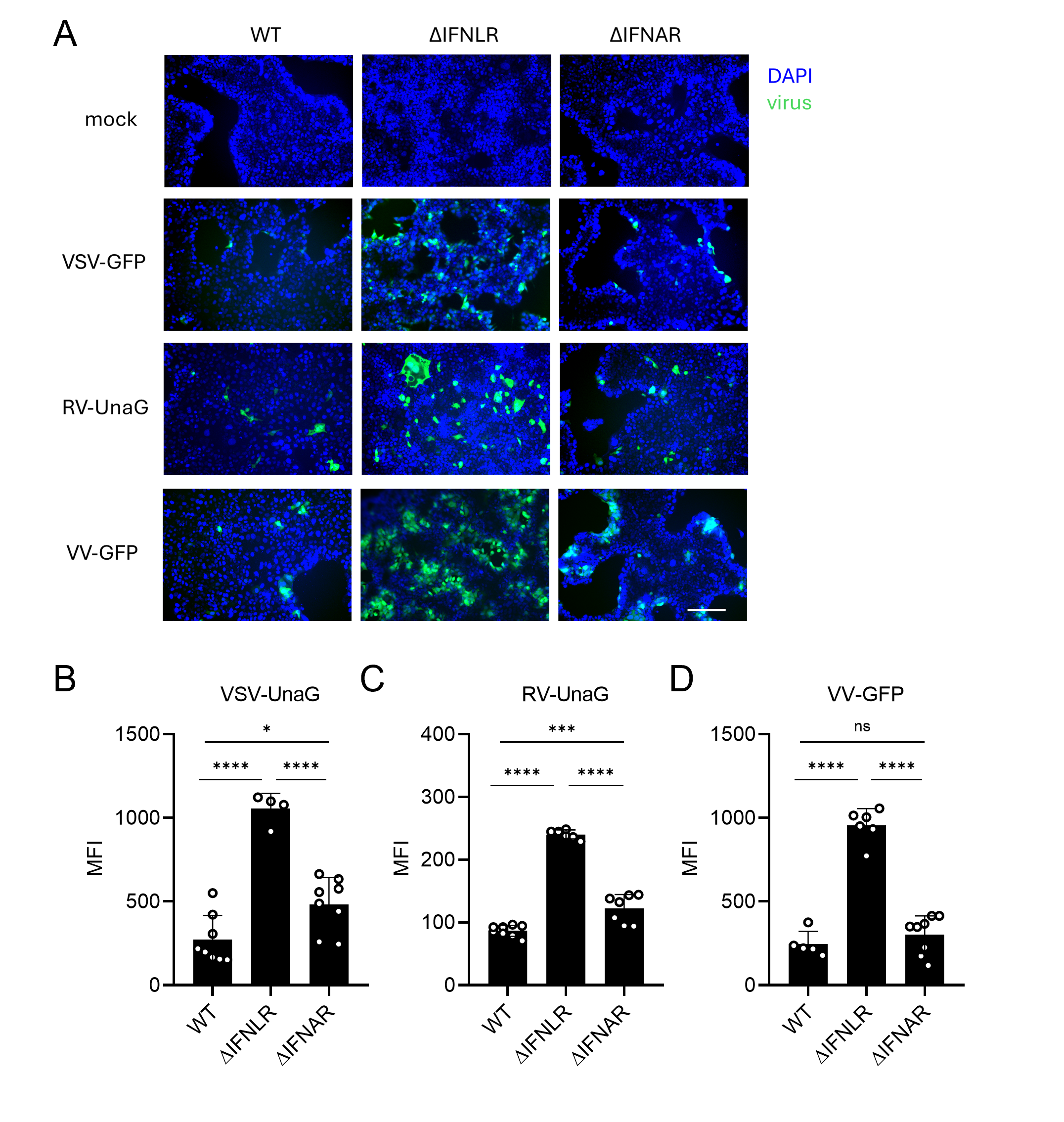

Supplement: S4 Fig — T84 WT, IFNAR KO, and IFNLR KO cells were seeded in 48-well plates and infected the following day. (A-D) Cells were infected with MOI (=1) (A) VSV-GFP for 7 hours, (B) RV-UnaG for 16 hours and (C) VV-GFP for 16 hours by live-cell microscopy. Nuclei were stained with Hoechst (blue), and infected cells are shown in green. (A) Representative images and (B-D) corresponding quantification are shown for each virus. Scale bar = 100 μm. Data represent ≥3 independent biological replicates. Statistical significance was determined by two-way ANOVA (*P < 0.05, ***P < 0.001, ****P < 0.0001, ns = not significant). Error bars represent standard deviation with the mean as the center. (TIF) [file ppat.1013857.s004.tif]

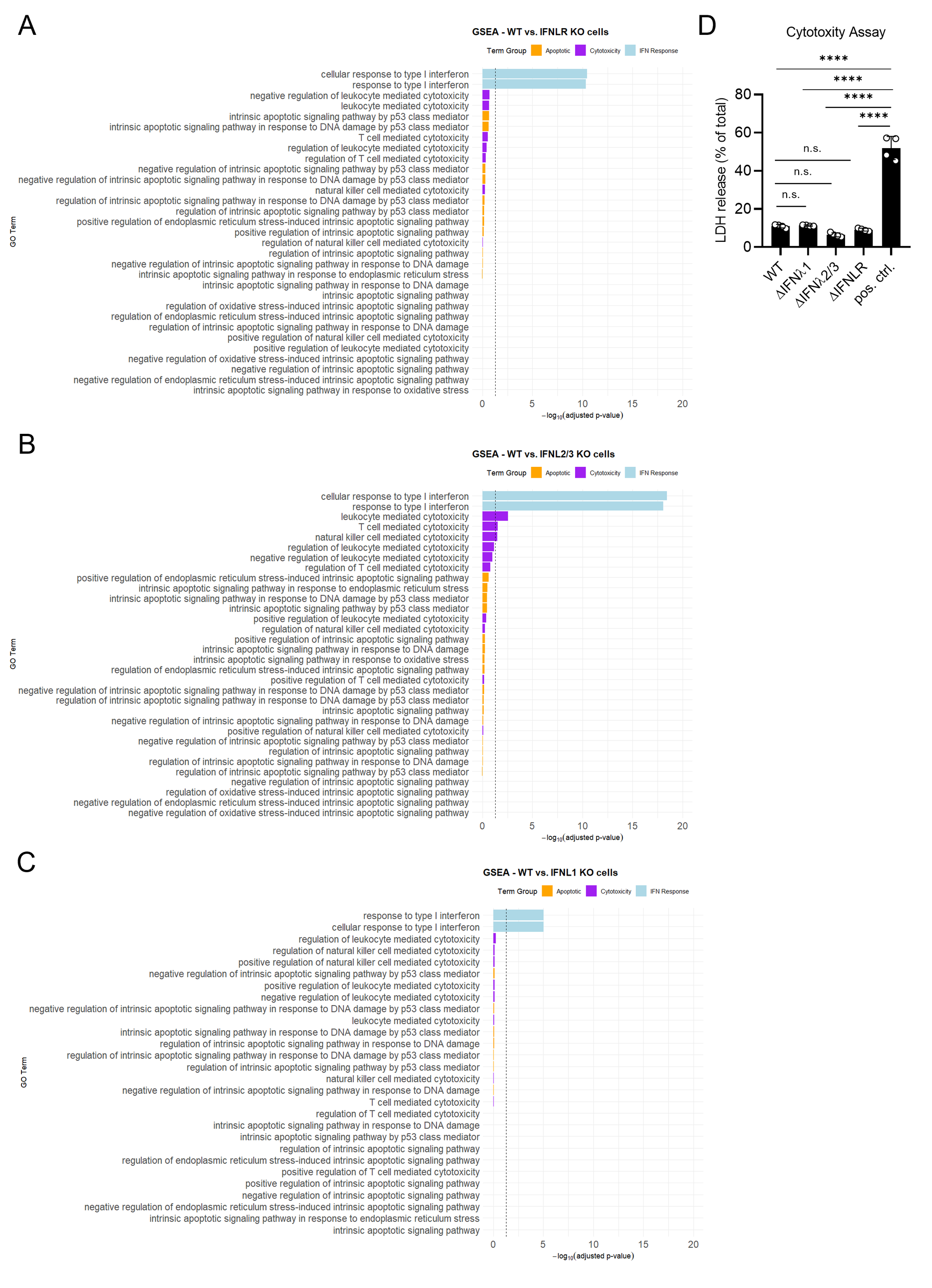

Supplement: S5 Fig — (A-C) Based on RNA-sequencing data, Gene Set Enrichment Analysis (GSEA) was performed on selected GO biological process terms associated with IFN response (blue), cytotoxicity (purple), and apoptotic pathways (orange). (A) Comparison of WT vs. IFNLR KO cells. (B) Comparison of WT vs. IFNλ2/3 KO cells. (C) Comparison of WT vs. IFNλ1 KO cells. (D) Cytotoxicity was assessed by measuring LDH release in WT, IFNλ1 KO, IFNλ2/3 KO and IFNLR KO cells. Positive control (pos. ctrl.) indicates cells treated with 50 μM PPMP to induce cytotoxicity. n ≥ 3 biological replicates. Statistical analysis was performed using ordinary one-way ANOVA. (P < 0.0001 ****, ns = not significant). Error bars represent standard deviation with the mean as the center. (TIF) [file ppat.1013857.s005.tif]

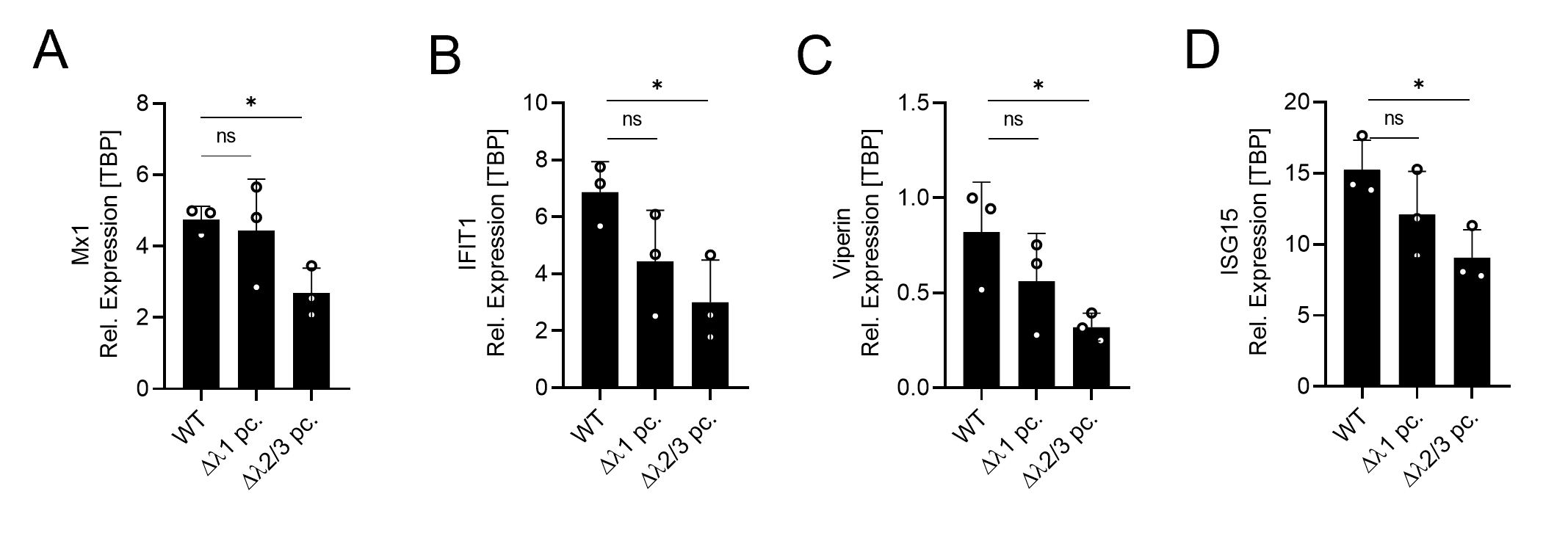

Supplement: S6 Fig — (A–D) qRT-PCR analysis of ISGs (Mx1, IFIT1, Viperin, and ISG15) in T84 WT cells, IFNλ1 KO polyclonal cells (pc.), and IFNλ2/3 KO polyclonal cells (pc.) at two days post-seeding. Relative expression was normalized to TBP. Data represent n ≥ 3 biological replicates. Statistical significance was determined using one-way ANOVA with multiple comparisons (P < 0.05 *, ns = not significant). Error bars represent standard deviation with the mean shown at the center. (TIF) [file ppat.1013857.s006.tif]

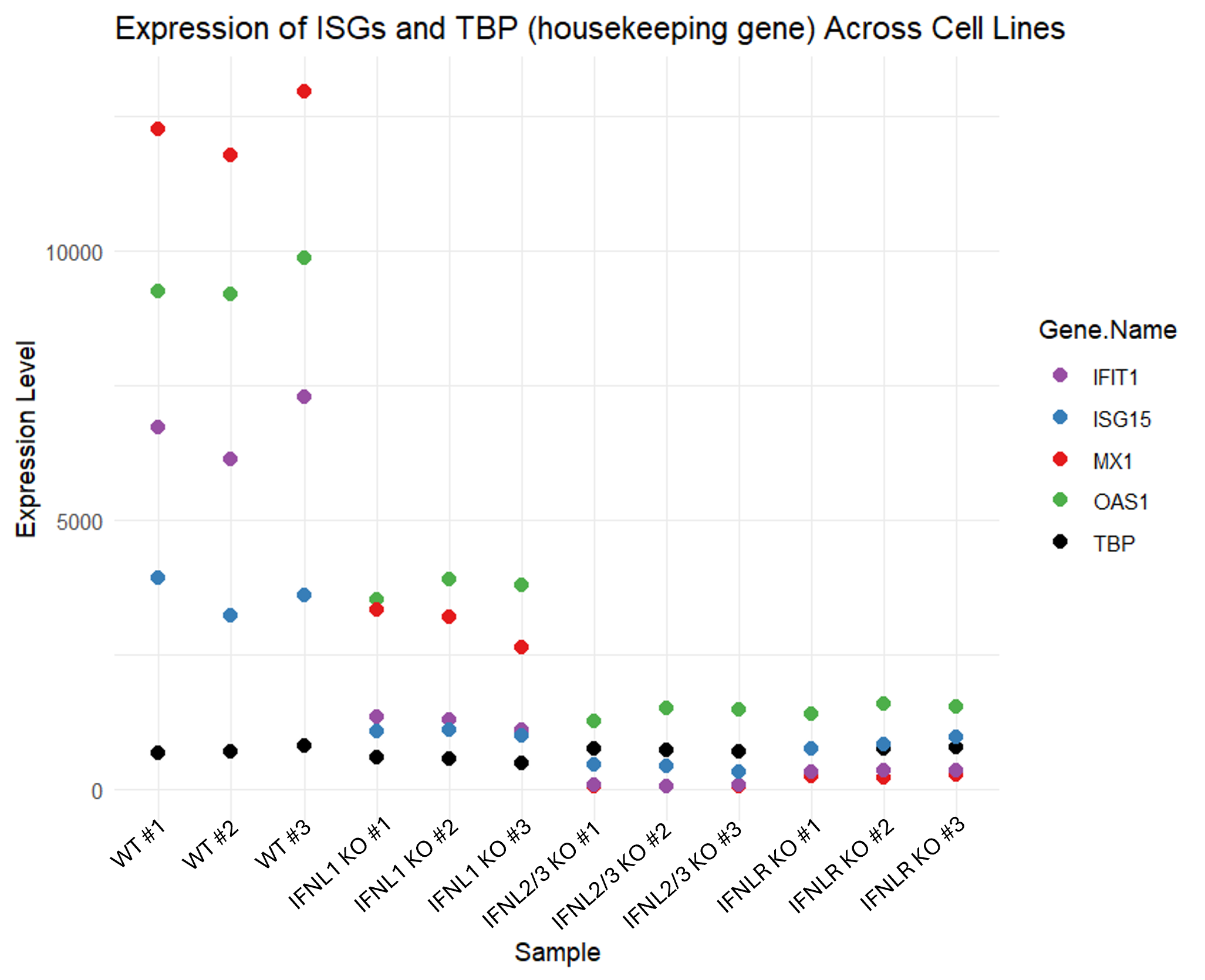

Supplement: S7 Fig — Expression levels of selected housekeeping gene (TBP) and ISGs (IFIT1, ISG15, MX1, OAS1) were analyzed across WT and KO cell lines using RNA-sequencing data. Each point represents an individual biological replicate. Expression levels are presented as normalized raw counts. (TIF) [file ppat.1013857.s007.tif]

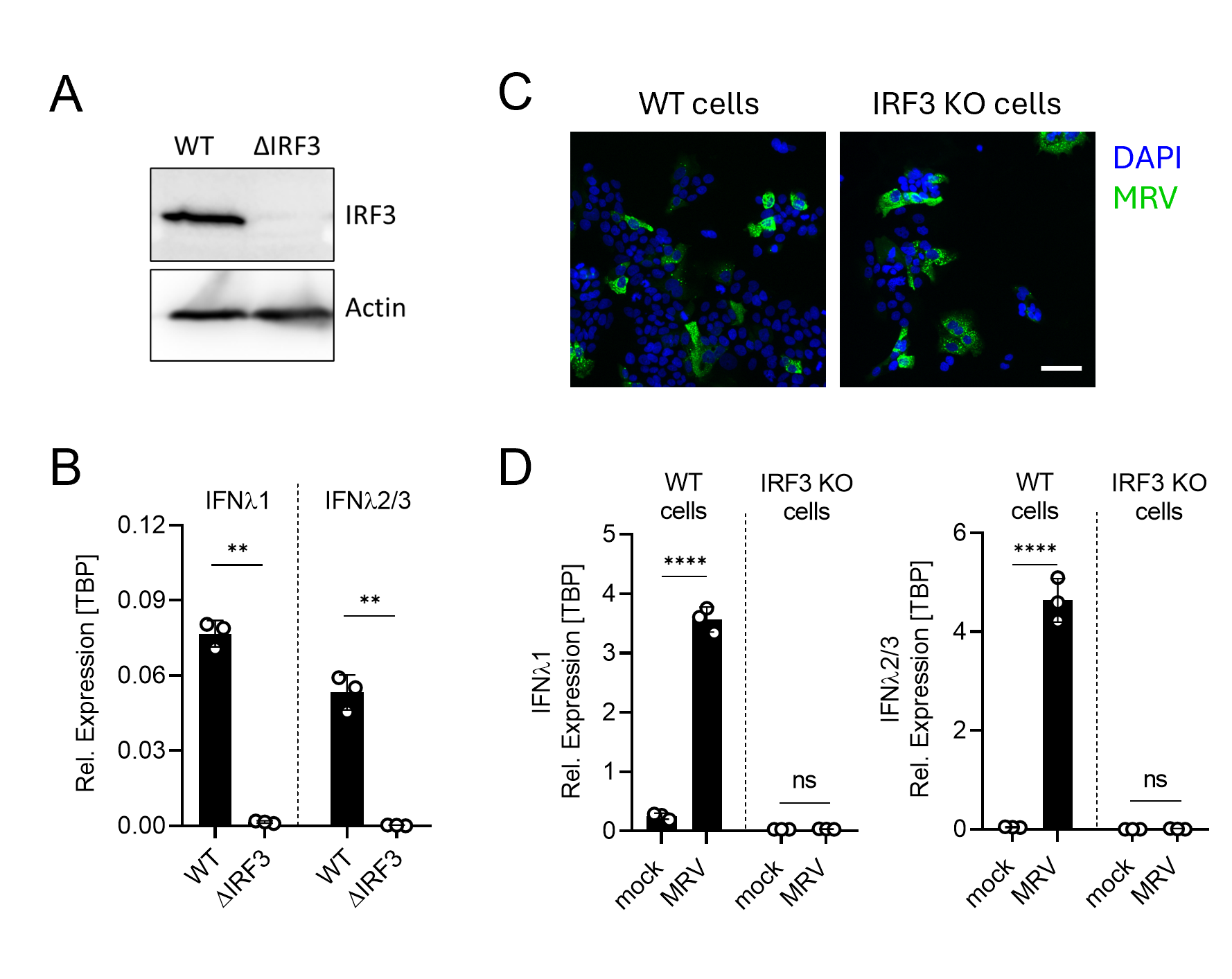

Supplement: S8 Fig — (A, B) T84 WT and IRF3 KO cells were seeded and harvested two days post-seeding to assess IRF3 expression. (A) IRF3 protein levels were analyzed by Western blot, with actin used as a loading control. Representative images shown. (B) Basal IFNλ1 and IFNλ2/3 expression levels were quantified by qRT-PCR in T84 WT and IRF3 KO cells. (C) T84 WT and IRF3 KO cells were seeded and infected with MRV the following day. MRV infection was assessed by immunostaining against the MRV µNS protein at 16 hpi. Representative images show nuclei (blue) and MRV-infected cells (green). Scale bar = 100 μm. (D) Same as C except MRV-induced IFNλ1 and IFNλ2/3 expression was quantified by qRT-PCR in T84 WT and IRF3 KO cells. Gene expression levels were normalized to TBP. Data represent n ≥ 3 biological replicates. Statistical significance was determined using (B) an unpaired t-test between WT and IRF3 KO cells and (D) by two-way ANOVA (P < 0.01 **, P < 0.0001 ****, ns = not significant). Error bars represent standard deviation with the mean as the center. (TIF) [file ppat.1013857.s008.tif]

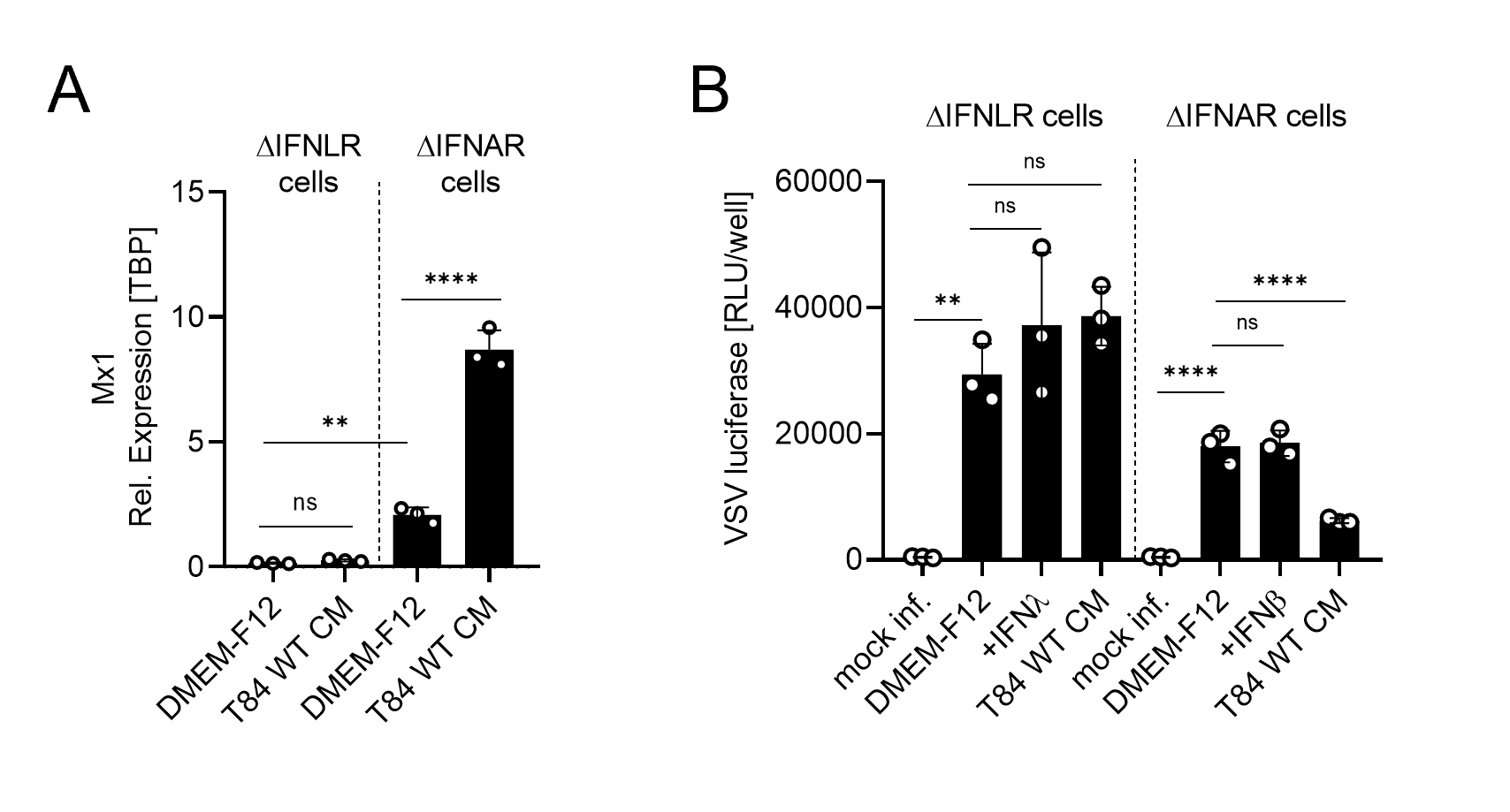

Supplement: S9 Fig — (A-B) T84 WT cells were seeded, and the media was changed the following day. Two days later, the cell supernatant was collected (referred to as conditioned media, CM) and used to treat T84 IFNLR KO cells (deficient in IFNλ signaling) and IFNAR KO cells (deficient in type I IFN signaling). Cells treated with culture media (DMEM-F12) served as controls. IFNLR KO cells were additionally pre-treated with recombinant IFNλ1–3 (100 ng/mL each) and IFNAR KO cells were additionally - pretreated with recombinant IFNβ (2000 IU/mL) for 24 hours prior infection as controls. (A) qRT-PCR analysis of the ISG Mx1 at 24 h post-treatment in IFNLR KO and IFNAR KO cells following CM exposure. Relative expression was normalized to TBP. (B) Following 24 h treatment with CM, cells were infected with VSV-Luc (MOI = 1). At 6 hpi, luciferase assays were performed to assess viral replication. Data represent n ≥ 3 biological replicates. Statistical significance was determined using two-way ANOVA (P < 0.01 **, P < 0.0001 ****, ns = not significant). Error bars represent standard deviation with the mean shown at the center. (TIF) [file ppat.1013857.s009.tif]

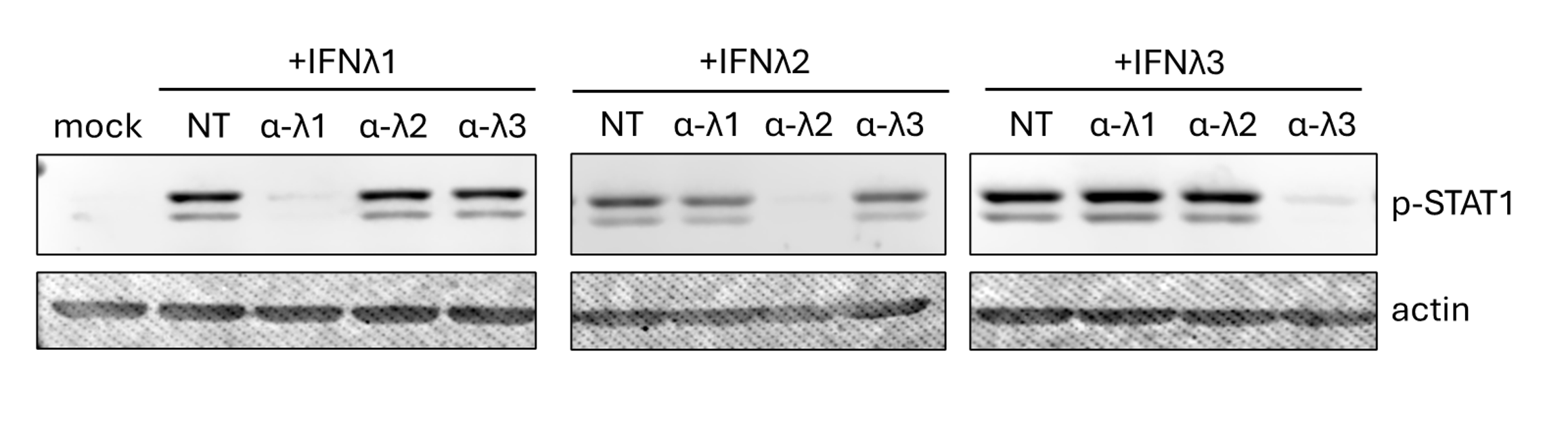

Supplement: S10 Fig — Recombinant IFNλ1, IFNλ2, or IFNλ3 (10 ng/mL each) was prepared in 250 μL of DMEM-F12 culture media containing 2.5 μL of the corresponding capture antibody and 2.5 μL of the corresponding detection antibody. Mixtures were incubated for 1 hour at room temperature and then immediately applied to T84 WT cells for 1 hour. Cells were harvested, and Western blot analysis of p-STAT1 was performed. Actin was used as a loading control. Representative images are shown. (TIF) [file ppat.1013857.s010.tif]
