# Supplementary material for: Basal IFNλ2/3 signaling is required for ISG expression and viral control in human intestinal epithelial cells
Source: PLoS Pathog. 2026 Jan 12;22(1):e1013857. doi: 10.1371/journal.ppat.1013857 (PMC12822948; doi:10.1371/journal.ppat.1013857)
Supplement: Attachment — Submitted filename: Response to Reviewers.docx [file ppat.1013857.s012.docx]

Dear Plos Pathogen editorial staff and reviewers,

We thank all three reviewers for their thorough evaluation of our manuscript and for their constructive feedback, which has substantially improved the quality and rigor of our work. We appreciate the time and expertise they devoted to evaluating our study. In response to their comments, we have substantially strengthened the manuscript with several key additions. First, we performed subtype-specific antibody neutralization experiments (new Figure 9) to definitively demonstrate that IFNλ2 and IFNλ3, rather than IFNλ1, drive basal antiviral protection independent of gene copy number effects. Second, we replaced the JAK inhibitor experiments with STING inhibitor (H151) studies (revised Figure 4) to selectively block basal IFNλ production while preserving virus-induced responses, directly demonstrating that basal IFNλ2/3 signaling establishes pre-infection antiviral readiness. Third, we validated our findings in polyclonal knockout populations (new Supplementary Figure 3) to exclude clonal artifacts. Fourth, we performed rescue experiments (new Figure 10) showing that chronic supplementation of IFNλ2/3 knockout cells with physiologically relevant levels of recombinant IFNλ2 or IFNλ3 restores basal ISG expression, STAT1 levels, and antiviral protection. Finally, we directly compared type I versus type III IFN contributions (new Supplementary Figure 4) and clarified culture conditions, demonstrating that type III IFNs are the predominant mediators of basal epithelial immunity. Together, these additions provide robust mechanistic evidence that basal IFNλ2/3 signaling maintains constitutive antiviral defense in intestinal epithelial cells. In addition, we have made requested editorial changes and uploaded requested documents. Please see below our point by point answer to reviewer comments. Reviewers comments are in **black** and our response is in **blue**.

Sincerely,

Megan Stanifer on behalf of all authors

**Reviewer #1:**

This well-written manuscript details studies investigating the relative contributions of IFNλ1 versus IFNλ2/3 to the recently-identified phenomenon of “basal” (not virus-induced) IFNL signaling in intestinal epithelial cells. Using an IEC cell line with various factors genetically disrupted, multiple viruses, drug treatment, and numerous experimental approaches including transcriptional analysis and supernatant transfers, the authors convincingly demonstrate that IFNλ2/3, but not IFNλ1, is important for basal ISG expression and initial viral regulation. Overall, experiments are well-conducted with appropriate controls for interpretation, and this is a nice study contributing to a growing field. However, there were a few points/questions that should be addressed.

We thank the reviewer for their careful evaluation of our work and for the constructive summary of our findings. We are pleased that the reviewer finds the manuscript well-written and agrees that our experimental framework, including the use of multiple genetically modified IEC lines, diverse viral models, pharmacological interventions, and complementary transcriptional and conditioned-media approaches, robustly supports the central conclusion that IFNλ2/3, rather than IFNλ1, drives basal ISG expression and early antiviral protection in intestinal epithelial cells. We appreciate the reviewer’s positive comments regarding the rigor of our controls and the overall contribution of this study to the emerging field of basal IFNλ biology. Below, we address each point and question in detail and outline the revisions and additional data incorporated in the revised manuscript.

**Major Points:**

(No Response)

**Minor Points:**

1. Lines 75-84: Would be appropriate to include findings from PMID 35137688 and 39893635 as part of this paragraph.

We thank the reviewer for this helpful suggestion. We have now incorporated the findings from PMID 35137688 and PMID 39893635 into this section of the introduction. These studies highlight the role of microbiota-driven, homeostatic IFNλ production in priming the intestinal epithelium for antiviral defense. We have integrated these findings alongside our recent demonstration of sterile, intrinsic basal IFNλ signaling to clarify that IFNλ can be expressed at baseline through both microbiota-dependent and microbiota-independent mechanisms. The revised paragraph now reflects these additional insights (line 101-108).

2. Lines 100-102: It would also be appropriate to include PMID 31462571 as a prior study demonstrating a role for IFNL cytokines that is equivalent to the role for the receptor in mice.

We thank the reviewer for this helpful suggestion. We have now incorporated PMID 31462571 into this section (line 128-129).

3. Is it possible to show the statistical comparisons between different doses and 0 in Figure 2? Perhaps with some sort of color scheme to demonstrate which comparisons are significantly different versus not?

We have now added the statistical comparisons between each IFNλ dose and the 0 ng/ml condition in Figure 2. These comparisons are indicated using a color-coded scheme.

4. Comment further on the loss of viral susceptibility in IFNλ1 KO cells in Fig 3?

This is a very interesting observation. As we see that IFNλ1 KO cells show similar or less ISGs than WT cells we do not think that this loss of susceptibility is purely mediated by basal immune signaling. From our other work we have found that IFNλ1 KO cells form very tight barriers suggesting that multiple mechanisms may be contributing to their restriction of virus infection. We find this very interesting and it is currently on going work in the lab.

5. Fig 5A: Can the authors please clarify what is contributing to the large separation between IFNλ2/3 and IFNLR KO lines? This is a somewhat surprising result.

IFNLR KO cells cannot respond to any IFNλ subtype, including IFNλ1, resulting in a complete loss of type III IFN signaling. In contrast, IFNλ2/3 KO cells retain IFNλ1-mediated signaling. Thus, the broader signaling deficit in IFNLR KO cells leads to more extensive transcriptomic changes, which likely accounts for their greater separation in the PCA plot. We anticipate that generating an IFNλ1/2/3 triple-knockout line would more closely phenocopy the transcriptional signature observed in IFNLR KO cells.

6. Fig 7: Were levels of the secreted basal IFNs (e.g. from WT cells) detectable by ELISA?

Unfortunately, the levels of secreted basal IFNλs in WT cell supernatant is below the detection limit of ELISA kits, and therefore we could not directly quantify it by this method. To address this limitation, we incorporated an additional functional assay in the revised manuscript (new Figure 10B). Specifically, we assessed the antiviral activity of WT conditioned media and compared it to titrated recombinant IFNλs. These experiments indicate that approximately ~1 ng/mL of IFNλ1 or IFNλ3, and ~5 ng/mL of IFNλ2, are sufficient to recapitulate the antiviral protection observed with WT conditioned media. This supports the conclusion that low-level, basal IFNλ production in WT cells is physiologically meaningful despite being below ELISA detection thresholds.

**Reviewer #2:**

 It is well established that IFN lambda is important for antiviral defense of epithelial barriers. A recent publication described basal IFNL production by epithelial cells upon reaching high density, but their effect on antiviral defense was not shown. This study extends the previous report of basal IFNL by determining how it impacts antiviral defense against diverse viruses and which IFNL subtypes may be most impactful in T84 cells. Using genetic knockouts, inhibitors, and supernatant transfer, the authors provide complementary evidence that basal IFNλ2/3 (but not IFNλ1) can boost antiviral immunity. Overall, the study was well written and clearly presented. However, there were several limitations that could be addressed to strengthen the most novel aspects of the study.

We thank the reviewer for their careful evaluation of our current and previous works and for recognizing the significance of our work in extending recent observations of basal IFNλ production by epithelial cells. We appreciate the reviewer’s positive comments regarding the clarity of the manuscript and the strength of the complementary approaches, including genetic knockouts, pharmacological inhibition, and supernatant-transfer assays, that collectively demonstrate the specific contribution of basal IFNλ2/3, but not IFNλ1, to epithelial antiviral priming. We also thank the reviewer for highlighting several limitations that, if addressed, would further strengthen the novelty and mechanistic insight of the study. In the revised manuscript, we have incorporated additional experiments, clarifications, and textual revisions to directly address these points. Below, we respond to each comment in detail.

**Major points:**

1. It is clear that the reported phenotype is not virus specific, but it Is perhaps more important to determine whether the phenotype is specific to T84 cells. Otherwise, this should be indicated as a limitation. T84 cells are a workhorse for mechanistic studies but are far from normal epithelial cells. The amount of basal IFNL seen here seems higher than expected for normal epithelial cells (often not detected in primary epithelial cells or biopsy from healthy intestine).

We appreciate the reviewer’s insightful comment regarding the potential cell line specificity of basal IFNλ expression. Indeed, we have recently addressed this question in our published study (Keser et al., 2025 - DOI: 10.1038/s44318-025-00539-5), which demonstrated that basal IFNλ expression is tightly regulated by epithelial cell density: high at high (H) density and low at low (L) density. In that study, we compared IFNλ expression across multiple epithelial models, including human intestinal (T84) and airway (Calu-3) epithelial cell lines, as well as primary ileum-derived organoids from two independent donors. In the figure below (previously published in Keser et al., 2025), IFNλ2/3 transcripts were readily detectable in all models, with expression in Ileum #1 comparable to T84 cells and Ileum #2 exhibiting approximately fourfold higher expression than T84 cells. These findings indicate that basal IFNλ2/3 expression is not restricted to T84 cells but represents a broader property of epithelial monolayers.

However, we acknowledge that due to technical limitations, we were unable to generate IFNλ knockout organoids, and therefore could not functionally assess the basal IFNλ phenotype in primary intestinal models. We have now clarified this point as a limitation in the revised manuscript (line 526-529).

2. Conclusions made about the relative importance of IFNλ2/3 vs IFNλ1 is weakened by the fact that IFNλ2 and IFNλ3 are two genes whereas IFNλ1 is a single gene. This is true for assays comparing mRNA abundance as well as comparison of IFNλ2/3 vs IFNλ1 knockout.

We thank the reviewer for pointing out that comparisons between IFNλ2/3 and IFNλ1 could be confounded by gene copy number. To directly address this concern, we performed a new series of experiments using antibody-mediated neutralization of individual IFNλ subtypes in conditioned media (now presented in Figure 9). This approach allows us to determine the functional contribution of each protein independent of gene number or transcriptional output, because all subtypes are present at their naturally produced levels and selectively depleted at the protein level.

Using subtype-specific neutralizing antibodies validated for recombinant IFNλ1, IFNλ2, and IFNλ3, we removed each IFNλ from T84-derived conditioned media and measured: JAK–STAT activation (pSTAT1), ISG induction (MX1), and functional antiviral protection (VSV-Luc assays) (Figure 9A).

These new data strengthen and clarify our conclusions: Neutralization of IFNλ3 or IFNλ2 caused the strongest loss of STAT1 phosphorylation, ISG induction, and antiviral protection (Figure 9B-D). In contrast, neutralization of IFNλ1 had only a modest effect and did not substantially reduce antiviral activity (Figure 9B-D). Importantly, combined neutralization of IFNλ2 + IFNλ3 did not exceed the effect of IFNλ2 or IFNλ3 alone, indicating that their contribution is redundant. Removal of all three IFNλ subtypes fully abolished signaling and antiviral protection, confirming that basal epithelial immunity in T84 cells relies almost entirely on IFNλ proteins (Figure 9B-D).

3. Conclusions from figure 4 are not really ruling out a role for virus-triggered responses as concluded in the text. In fact, I would conclude that there is a role for IFNλ2/3 triggered by the virus as indicated by the effect of the inhibitor in the IFNλ1 knockouts (where virus-triggered IFNλ2/3 may be playing a role). Additionally, the JAK inhibitor is going to impact Type I IFN signaling so this experiment may be suggestive at best.

We thank the reviewer for this comment. We fully agree that viral infection can further induce IFNλ2/3 expression and thereby contribute to antiviral protection. Indeed, the residual antiviral activity observed in IFNλ1 knockout cells, which was abolished upon JAK inhibition, is consistent with the activation of virus-induced IFNλ2/3 signaling. Our intent in this figure, however, was not to exclude a virus-triggered component, but rather to demonstrate that basal IFNλ2/3 signaling, present prior to infection, is essential for establishing an antiviral-ready state as ruxolitinib had no further effect in IFNλ2/3 KO or IFNLR KO cells, both of which were already maximally permissive, supporting that basal IFNλ2/3 signaling establishes a crucial pre-infection antiviral state.

However, we appreciate that the current experiment could lead to misinterpretation regarding the relative contribution of basal versus virus-induced signaling, and it does not directly demonstrate the antiviral function of basal IFNλs. To address this concern, we designed a new set of experiments to selectively inhibit basal IFNλ expression without interfering with the virus-induced response. As shown in our recent study (Keser et al., 2025 - DOI: 10.1038/s44318-025-00539-5), basal IFNλ production in T84 cells is driven by cGAS–STING sensing of mitochondrial DNA at steady state. We therefore used the STING inhibitor H151 to acutely suppress basal IFNλ expression in T84 WT, IFNLRKO, IFNλ1 KO, and IFNλ2/3 KO cells prior to infection with RNA virus, VSV-Luc (Figure 4). H151 effectively reduced basal IFNλ levels by qRT-PCR while leaving the VSV-induced IFNλ response intact (Figure 4A, B, E, G). H151 markedly enhanced infection in WT cells, whereas it had no effect in IFNLR KO cells, demonstrating that STING-dependent basal IFNλ signaling is the major source of intrinsic antiviral protection (Figure 4C, D). Importantly, inhibition of basal IFNλ2/3 in IFNλ1 KO cells significantly increased viral replication, while inhibition of basal IFNλ1 in IFNλ2/3 KO cells had no effect (Figure 9F, H). This highlights the dominant role of basal IFNλ2/3, rather than IFNλ1, in maintaining basal antiviral defense.

Finally, to directly compare basal type I versus type III IFN contributions, we assessed the susceptibility of T84 WT, IFNLR KO, and IFNAR KO cells to VSV-GFP, RV-UnaG, and VV-GFP infection (Sup. Figure 4). Loss of IFNAR caused only a modest increase in infection, whereas IFNLR deficiency markedly enhanced infection across all viruses tested (Sup. Figure 4). Moreover, conditioned media from WT cells induced ISGs and conferred antiviral protection in IFNAR KO cells but not in IFNLR KO cells, further confirming that type III IFNs are the predominant antiviral mediators in intestinal epithelial cells (Sup. Figure 9).

Together, these additional data directly address the reviewer’s concern and demonstrate that although virus-induced IFNλ2/3 contributes to antiviral defense, the essential component of intrinsic epithelial protection is the STING-driven basal IFNλ2/3 axis, which establishes a pre-infection antiviral state.

4. There is a convincing phenotype of the IFNλ2/3 knockout in diminished response to IFN treatment, but if the authors’ model is correct then this should be rescued by extended culture with low levels of basal IFNλ2/3 prior to a subsequent treatment with the dose used in fig. 6D. The supernatant transfer assay is strong, but is not definitive because the effect may be due to combination of factors missing from the sup of IFNλ2/3 ko. A definitive experiment would be to add low levels of recombinant IFNλ2 or IFNλ3 and show that this rescues the effect. This could address another caveat of the genetic studies which is that the knockout of two genes IFNλ2 and IFNλ3 is being compared to the knockout of a single gene IFNλ1. Rescue/add-back studies with single types at low/physiologal levels (whatever present in sup transfer) could further distinguish the activities of the subtypes if there are any.

We thank the reviewer for this excellent suggestion. To directly test whether low, physiologically relevant levels of IFNλ2 or IFNλ3 can rescue the phenotype observed in IFNλ2/3 knockout cells, we performed the additional experiments shown in new Figure 10 (and shown below). Because IFNλ2/3 protein levels are below the detection limit of ELISA, we first used a bioassay-based calibration to identify the range of recombinant IFNλ2 and IFNλ3 concentrations that mimic the antiviral protection conferred by conditioned medium from T84 WT epithelial cells (Figure 10A,B). We found that approximately 1 ng/mL IFNλ3 and 5 ng/mL IFNλ2 reproduce the antiviral protection conferred by WT supernatant, suggesting that these concentrations represent physiologically relevant basal levels (Figure 10A and B).

Using these optimized doses, we cultured IFNλ2/3 KO T84 cells with recombinant IFNλ2 and/or IFNλ3 for two weeks (Figure 10C). This extended pre-conditioning significantly increased ISG expression in transcript and protein level (Figure 10D and E) and markedly improved antiviral protection against VSV-Luc infection (Figure 10F). Importantly, co-treatment with both IFNλ2 and IFNλ3 did not show additive effects in ISG expression or virus replication that was observed in IFNλ3 treatment alone, suggesting their overlapping functions (Figure 10D and F).

Furthermore, chronic supplementation with IFNλ2 or IFNλ3 restored total STAT1 protein levels in IFNλ2/3 knockout cells and improved STAT1 phosphorylation following acute IFNλ1–3 stimulation (Figure G and H). These results demonstrate that the impaired JAK–STAT signaling observed in IFNλ2/3 knockout cells is reversible and directly attributable to the absence of basal IFNλ2/3 signaling rather than secondary consequences. Importantly, this recovery was achieved using single-subtype supplementation, allowing us to functionally distinguish the individual contributions of IFNλ2 and IFNλ3. Interestingly, this was not reflected in induced ISG levels with second acute IFNλ1–3 stimulation. This was unexpected; however, this might be explained by the fact that restoration of STAT1 abundance and phosphorylation does not immediately translate into a proportional increase in downstream ISG transcription. Acute ISG induction is shaped not only by STAT1 availability, but also by additional regulatory layers, such as chromatin accessibility, STAT1/STAT2 nuclear dynamics, and the refractory or desensitized state induced by prior IFN exposure. Thus, although chronic IFNλ2/3 supplementation reestablishes the core signaling machinery, the transcriptional landscape may remain partially refractory, limiting further ISG upregulation upon a secondary acute IFN stimulus.

Overall, these data strengthen our conclusion that basal, low-level IFNλ2/3 signaling is essential for maintaining basal ISG expression and antiviral readiness in epithelial cells, and that the phenotype in IFNλ2/3 KO cells is directly attributable to the loss of this basal signaling rather than secondary factors in the conditioned media.

**Minor points:**

1. Line 44 “IFNL4 has been detrimental to humans' evolution…” is a pretty strong statement. It seems to have had some sort of benefit as it is highly represented in African populations (<https://doi.org/10.1371/journal.pgen.1004681>) so better to not make this conclusion that it is solely detrimental.

We thank the reviewer for this insightful comment. We agree that stating IFNλ4 was “detrimental” is too strong and does not fully capture its population-specific evolutionary dynamics. We have therefore revised the text to more accurately reflect the complex and context-dependent selection on IFNλ4 (Line 66-69).

2. GSEA in supplementary figure 4 is not very convincing and has no pvalue. Not sure it adds anything to the genes shown in volcano plot and heatmap in fig 5.

We appreciate the reviewer’s feedback regarding the GSEA analysis in Supplementary Figure 4. Given that the enrichment results did not provide additional insight beyond the volcano plot and heatmap in Fig. 5, we have removed this analysis from the revised manuscript.

3. Some of the text for RNAseq figures (5 and S5) is tiny and impossible to read. Consider reducing the number of labels or pathways shown and increasing fontsize

In the revised manuscript, we have increased the font size and reduced the number of pathway labels to improve clarity and ensure that all text is easily readable.

4. Line 414-417. Rodents have IFNλ1 or an IFNλ1-like gene but it is a pseudogene, so I don’t this this statement about its primate specificity is entirely accurate?

We thank the reviewer for this helpful clarification. IFNλ1 is indeed a pseudogene in mice. We have revised the text to more accurately state that the functional IFNλ1 gene is largely restricted to primates (line 509-513.

**Reviewer #3:**

In this study, Keser et al dissect the distinct biological functions of IFNλ1 and IFNλ2/3 in conferring antiviral protection in T84 intestinal epithelial cell lines. This question is highly relevant and could uncover specialized mechanisms of mucosal antiviral defense. Using genome-edited cells, they demonstrate viral infection can induce IFNλ1 and IFNλ2/3, and these cytokines have equivalent antiviral activity. However, there seems to be a predominant requirement for basal IFNλ2/3 expression in inducing low-level expression of ISGs that can protect cells against viral infection. While the study provides novel insights, there are a few technical limitations that preclude a firm conclusion that basal ISG expression driven by IFNλ2/3 is the underlying protective mechanism. Further positioning the study in context with prior work from this group and others would also help readers better appreciate how these results fit within the broader framework of IFNλ biology, including the potential influence of culture conditions and cell polarization on basal IFNλ expression and ISG induction.

We thank the reviewer for their thoughtful assessment and for highlighting the importance of dissecting IFNλ1 versus IFNλ2/3 biology in mucosal antiviral defense, as well as for recognizing our prior work in this area. In the revised manuscript, we have incorporated additional experiments specifically designed to strengthen our conclusion that basal ISG expression driven by IFNλ2/3 underlies the observed protective mechanism. We have also expanded the Methods section to provide more detail on culture conditions and revised the Discussion to better address current limitations and outline future directions. We believe these revisions address the reviewer’s concerns and substantially improve both the clarity and rigor of the study.

**Major Points:**

1. While the Ruxolitinib experiments are informative, they do not fully resolve the need for basal IFNλ2/3 signaling as the underlying mechanisms for increased viral susceptibility of IFNλ2/3 KO cells. Because the lines were single-cell cloned and already exhibit reduced STAT1/STAT2/IRF9 and blunted inducibility, clonal artifacts or loss of signaling components could explain the phenotype. I recommend the authors validate the phenotype in a pooled (polyclonal) edited population or in multiple independent KO clones and show that transient depletion (siRNA or neutralizing antibody) in parental cells reduces basal ISGs expression and conditioned-media activity to demonstrate that ongoing basal ligand signaling, rather than clone-specific effects or secondary loss of signaling machinery, is responsible for the observed phenotypes.

We thank the reviewer for this insightful comment and fully agree that validation beyond single-cell clones is crucial to exclude clonal artifacts and confirm that the observed phenotype truly reflects loss of basal IFNλ2/3 signaling. To address this, we performed two complementary sets of validation experiments.

First, we generated freshly edited T84 polyclonal knockout populations targeting IFNλ2/3 and IFNλ1. As shown in newly added Supplementary Figure 3, the IFNλ2/3 KO polyclonal population exhibited markedly reduced basal ISG expression and increased viral susceptibility compared to both WT and IFNλ1 KO polyclones (Sup. Figure 3D-G). These results confirm that the phenotype is reproducible across independent knock out events and is not attributable to clonal variation or secondary mutations affecting downstream signaling components.

Second, to acutely disrupt ongoing basal IFNλ signaling in parental cells, we utilized antibodies raised against IFNλ1, IFNλ2, and IFNλ3, and we validated their specificity using recombinant IFNλ treatment (Sup. Figure 10). The conditioned media from WT cells was pre-incubated with the respective neutralizing antibodies for 1 hour prior to application to naïve T84 or IRF3-deficient reporter cells (Figure 9A). Results showed that neutralization of IFNλ2 and IFNλ3 significantly reduced STAT1 phosphorylation (Figure 9B), downregulated ISG expression (Figure 9C), and substantially increased viral infection in both assays (Figure 9D). In contrast, neutralization of IFNλ1 had less pronounced effect, consistent with our prior findings that IFNλ2/3 are the predominant basal ligands. Importantly, co-neutralization of IFNλ2 and IFNλ3 did not produce additive effects, reflecting their functional redundancy (Figure 9B-D).

Together, these data robustly validate that the antiviral and ISG phenotypes observed in IFNλ2/3 KO cells are not due to clonal artifacts or loss of signaling capacity, but rather stem from the absence of ongoing basal IFNλ2/3 ligand production and signaling that maintains epithelial antiviral readiness in the steady state.

2. The authors have left the culture conditions undefined. Prior work shows that basal IFNλ2/3 induction is highly context-dependent (confluency, polarization, and Hippo-cGAS-STING signaling). Could the authors please clarify how the cells were cultured? Are the growth kinetics across clones equivalent? If not, is this a function of IFNλ signaling? Additional studies are needed to elucidate the expression levels of IFNλ1 and IFNλ2/3 under low and high confluency, as well as their induction during viral infection, to better understand the mechanisms that influence their expression, basal ISG expression, and the distinct role of IFNλ after viral challenge.

We thank the reviewer for this thoughtful comment and for acknowledging our recent work. In this study, all experiments were performed using T84 monolayers maintained at 70–80% confluence, a stage at which cells begin to establish a monolayer but remain permissive to viral infection (see below). Based on our observations, once T84 cells form an intact polarized monolayer reaching >100% confluence, they exhibit strong resistance to virus infection (unpublished data below). For this reason, we selected this intermediate confluency window (~70-80%) to ensure that basal IFNλ signaling is active while preserving viral susceptibility, both of which are essential for the experimental readouts in this work. This has been added to the methods section.

Growth kinetics across all clonal lines, including WT, IFNλ1 KO, and IFNλ2/3 KO, were comparable, and we did not observe differences attributable to the presence or absence of basal IFNλ signaling (data below. left). Consistent with our prior work, basal IFNλ1 and IFNλ2/3 expression increased gradually as cells approached confluence, and the two subtypes exhibited only modest differences in magnitude (data below, right).

We agree with the reviewer that a more detailed dissection of IFNλ expression dynamics under low versus high confluency, as well as during viral infection, would provide deeper mechanistic insight. Although these analyses were beyond the scope of the current study, they represent important future directions that will help clarify how epithelial differentiation state and cell density shape basal ISG regulation and the distinct roles of IFNλ subtypes during viral challenge.

3. If cells can make both IFNλ1 and IFNλ2/3 during infection, and all three cytokines have similar activities in T84 cells, it is unclear why the authors propose that only IFNλ2/3 would confer protection against infection and basal ISG expression in T84 cells. Is this also the case in other intestinal epithelial cell lines and primary cells? Are these phenotypes conserved in other IFNλ-responsive epithelial cell types?

We thank the reviewer for this important question. Although T84 cells express IFNλ1 and IFNλ2/3 at comparable basal levels and all three cytokines can exert a robust antiviral protection when given exogenously (Figure 2), our genetic, neutralization, and inhibitor experiments show that basal IFNλ2/3 dominate basal ISG expression and antiviral protection (Figure 3-9). Deletion or neutralization of IFNλ2 and/or IFNλ3 abolishes basal ISGs and increases viral susceptibility, whereas loss or inhibition of IFNλ1 has negligible effect (Figure 3-9). Consistent with this, blocking basal IFNλ signaling with the STING inhibitor H151 in IFNλ1 KO cells significantly increased viral replication, while blocking IFNλ1 in IFNλ2/3 KO cells did not, supporting a dominant role for basal IFNλ2/3 (Figure 4).

Importantly, these findings do not contradict the similar antiviral potency of all IFNλ subtypes at high (induced or exogenous) doses; rather, they reveal a hierarchy at physiological (basal) levels in which IFNλ2/3 has the strongest role in maintaining basal JAK–STAT signaling, with minimal contribution from basal IFNλ1. We acknowledge that we could not yet validate this phenotype in ileum-derived primary organoids due to current technical barriers in generating targeted knockouts in this system, this is now acknowledged as a limitation in revised manuscript (line 525-528). Testing whether this hierarchical contribution of IFNλ subtypes is conserved across additional epithelial models will be an important direction for future work.

**Minor Points:**

1. Supplementary figure 5: Consider plotting only significant terms to make the data more accessible.

In the revised manuscript, we have increased the font size and reduced the number of pathway labels to improve clarity and ensure that all text is easily readable.
